# Acute and lifelong exercise modulate the tumorigenic potential of human lung cancer cells and their susceptibility to cisplatin

**DOI:** 10.1113/EP093973

**Published:** 2026-07-25

**Authors:** Carlos M. Soares, João P. Moura, Leonardo M. R. Ferreira, Ana Pedrosa, Pedro Filipe, Luís Rama, Ana M. Teixeira, Ana M. Urbano

**Affiliations:** ^1^ CIPER, Faculty of Sport Sciences and Physical Education University of Coimbra Coimbra Portugal; ^2^ Molecular Physical Chemistry R&D Unit (QFM‐UC) University of Coimbra Coimbra Portugal; ^3^ CI‐ISCE Higher Institute of Educational Sciences of the Douro Penafiel Portugal; ^4^ Department of Life Sciences University of Coimbra Coimbra Portugal; ^5^ Department of Pharmacology and Immunology Medical University of South Carolina Charleston South Carolina USA; ^6^ Hollings Cancer Center Medical University of South Carolina Charleston South Carolina USA; ^7^ University Hospitals of Coimbra University of Coimbra Coimbra Portugal; ^8^ LAQV/Requimte, Center of Investigation in Environment, Genetics and Oncobiology (CIMAGO) University of Coimbra Coimbra Portugal

**Keywords:** cancer risk, cell migration, cell proliferation, cytokine, exercise‐conditioned human serum, susceptibility to chemotherapy

## Abstract

The association between higher levels of physical activity and lower cancer risk and mortality is well established. However, a causal link is yet to be proven. Recent studies showed a decrease in the proliferation rates of cultured human cancer cells when the human serum used to stimulate them was conditioned by acute exercise. Here, we tested the hypothesis that serum mediates some of the putative benefits of exercise on cancer through alterations to the growth pattern and susceptibility to chemotherapy agents of cancer cells. To this end, human non‐small cell lung cancer cells were exposed to serum, collected before or after an acute exercise intervention, from two cohorts that differed significantly in their levels of physical activity and, accordingly, cardiorespiratory fitness, but were otherwise identical (non‐exercisers and master athletes). Serum levels of glucose, lipids, albumin, C‐reactive protein and cytokines were determined, and the impact of the serum responses to acute and lifelong exercise on cancer cell growth pattern and susceptibility to cisplatin were analysed. We found that acute exercise decreased the proliferation rate of the cells, yet shortened the lag phase of the cells after detachment, whereas lifelong exercise had the opposite effects. Significantly, we showed, for the first time, that lifelong exercise increased susceptibility to a chemotherapy agent, which might contribute to the decreased cancer mortality rates found among those cancer patients who exercise regularly. Similar to the cellular effects, changes to serum cytokine levels (several of them linked to the senescence‐associated secretory phenotype) depended on whether serum was conditioned by acute exercise or by lifelong exercise.

## Abstract (highlights)

- **What is the central question of this study?**
  Do serological responses to exercise provide anticancer benefits, e.g., an improved response to chemotherapy?
- **What is the main finding and its importance?**
  The main finding was a substantially lower half‐maximal cytotoxic concentration value for cisplatin against A549 lung cancer cells when the serum used to stimulate them was conditioned by lifelong exercise, which is in line with the improved response to chemotherapy observed, in epidemiological studies, amongst cancer patients who exercise.

## 1 INTRODUCTION

Cancer is one of the largest health problems in the world, accounting for every sixth death globally. And yet, a large percentage of cancer cases and cancer deaths could be prevented simply by eliminating or reducing exposure to environmental and occupational risk factors (Ádám et al., 2023; Katzke et al., 2015; Lewandowska et al., 2019; Urbano et al., 2008, 2012) and by adopting healthier lifestyles, e.g., quitting smoking, maintaining a healthy weight, reducing alcohol consumption and being more active (Friedenreich et al., 2010; Garcia et al., 2023; McTiernan, 2008).

The evidence associating physical inactivity and increased cancer risk and mortality is robust. For instance, a very large pooled analysis from 2016 involving 1.44 million participants showed that the risk for 13 out of 26 common types of cancer was lower for those participants who self‐reported engagement in higher levels of moderate or vigorous intensity leisure‐time physical activity than for those participants who self‐reported lower levels of engagement (Moore et al., 2016). More recently, another meta‐analysis involving >30 million participants yielded an inverse dose–response association between non‐occupational physical activity and cancer‐related disease and mortality (Garcia et al., 2023). The evidence for a positive association between exercise and reduced cancer mortality has been strengthened very recently by a randomized trial, showing that a 3‐year structured exercise programme initiated soon after adjuvant chemotherapy significantly extended disease‐free survival, with findings consistent with longer overall survival (Courneya et al., 2025).

Despite the amount and strength of the evidence discussed above, these associations are not proof that higher levels of physical activity reduce cancer risk and/or improve cancer management. For instance, it cannot be ruled out that the lower cancer risk and mortality rates found amongst active people is a consequence of other concurrent healthy habits or underlying genetic predispositions that might be present in active people. Establishing a causal relationship would constitute a strong inducement for the >30% of the worldwide population who do not attain the levels of physical activity recommended by the World Health Organization (WHO) (Bull et al., 2020; Elgaddal *et al.*, 2022) to significantly reduce the amount of time spent in sedentary occupations.

It has been argued that the observed reduction of cancer risk and mortality associated with physical activity is primarily an indirect effect of weight loss, especially in terms of adiposity. The fact that an association was found between excess body fat and a higher risk for a large number of cancer types (Avgerinos et al., 2019; Lauby‐Secretan et al., 2016) lends some support to this view. Lowering the number of adipose cells and/or their size reduces the secretion and, hence, the circulating levels of several biomarkers of cancer risk, such as sex hormones, metabolic hormones (e.g., leptin and resistin), insulin‐like growth factors and pro‐inflammatory cytokines, also decreasing macrophage infiltration into adipose tissue, ultimately lowering systemic inflammation (Avgerinos et al., 2019; Michailidou et al., 2022; Tilg et al., 2025). In addition, obesity weakens the anti‐tumour responses of natural killer cells, thus compromising immunosurveillance and the elimination of cancer cells (Michelet et al., 2018). Nonetheless, the fact that the inverse associations between physical activity and cancer risk were unaffected by body size in 10 of the above‐mentioned 13 types of common cancers (Moore et al., 2016) suggests that mechanisms independent from weight loss contribute to the putative beneficial effects of physical activity.

There is increasing evidence that the growth patterns of cultured human cancer cells are altered when the human serum used to stimulate them is conditioned by exercise. Although these in vitro studies are still sparse and vary considerably in all aspects of study design, meta‐analyses do suggest that, in comparison to baseline serum, serum conditioned by an acute exercise intervention diminishes the viability of cultured cancer cells, with a large overall effect size that increases with the intensity of the exercise (Brown et al., 2021; Orange et al., 2020; Soares et al., 2021). In this exploratory study, we exposed A549 human non‐small cell lung cancer cells to human sera obtained before or after acute exercise and analysed the impact of the transient serum responses to the intervention on the ability of the sera to stimulate cancer cell proliferation, on the plating efficiency of the cells, here used as a metric for reproductive potential (assessed previously in only one study), lag phase (not previously reported), cell migration (not previously reported) and susceptibility to chemotherapy (not previously reported). We recruited two distinct cohorts with varying physical activity levels, namely volunteers who did not meet the WHO physical activity guidelines (non‐exercisers) and volunteers who had exercised for most of their adult lives (master athletes). This strategy allowed us to examine not only how lifelong physical activity levels influence the serum‐mediated impact of acute exercise on these cellular parameters, but also how these same parameters are affected by permanent serum changes induced by lifelong exercise. The latter was assessed by comparing the behaviour of cancer cells stimulated with baseline serum (i.e., collected at rest) from lifelong exercisers with that of cancer cells stimulated with baseline serum from non‐exercisers.

## 2 MATERIALS AND METHODS

### 2.1 Ethical approval

The study was approved by the Ethics Committee of the Faculty of Sports Sciences and Physical Education of the University of Coimbra (reference CE/FCDEF‐UC/00062013). All procedures conformed to the *Declaration of Helsinki* (World Medical, 2013) and with data protection and security regulations (Harriss & Atkinson, 2015), except for registration in a database. At the time of recruitment, the intervention and potential risks were explained to the participants who all gave written informed consent before their inclusion in the study.

### 2.2 Participants

Two age‐matched cohorts were recruited, each consisting of 13 Caucasian healthy male volunteers aged 40–63 years: a cohort of non‐exercisers [individuals who self‐reported not having met, in the 20 years that preceded their recruitment, the WHO guidelines on physical activity for health (Bull et al., 2020)] and a cohort of master athletes, all of them self‐reported experienced competitors who had been training and competing for ≥20 years (average training duration: 40.55 ± 15.56 years; average weekly training volume during the recruitment season: 6.21 ± 4.74 h/week) in their sports modality (endurance sports, combat sports, rowing, rugby and padel) at the time of recruitment. Height and body weight were determined using a Harpenden 98.603 stadiometer (Holtain, Crosswell, UK) and Seca 770 electronic personal scales (Seca, Hamburg, Germany), respectively. Body mass composition was assessed using an InBody 770 tetrapolar bioelectrical impedance analyser (InBody, Cerritos, CA, USA) (Brewer et al., 2021). Basal metabolic rate was calculated by the analyser according to Cunnigham's equation [basal metabolic rate (in kilocalories per day) = 370 + 21.6 × fat‐free mass (in kilograms)] (Cunningham, 1991). Exclusion criteria included smoking, having donated blood in the 3 months that preceded the intervention, any evidence of chronic or inflammatory diseases, having had an infectious disease ≤6 weeks prior to the exercise intervention, and taking supplementation or medication. Participants were asked to maintain their lifestyle routines and to avoid caffeine, exercising and partaking in sports activities in the 24 h preceding the exercise intervention.

### 2.3 Acute exercise intervention

All participants performed a single bout of maximal incremental voluntary aerobic protocol (Åstrand, 1964), here used as both a physiological assessment and an acute exercise intervention. This approach ensured that each participant, regardless of their initial fitness level, reached their individual physiological limits, providing a standardized, high‐intensity systemic stimulus. The protocol, which was preceded by a 5 min warm‐up of moderate cycling, was carried out on an electromagnetically braked Excalibur Sport bicycle ergometer (Lode BV, Groningen, the Netherlands). Throughout the protocol, expired gases were analysed using a Quark CPET breath‐by‐breath automated gas‐analysis system (COSMED, Rome, Italy), which was also used for continuous telemetric determination of heart rate. Initially set at 75 W, the power output was increased by 25‐W increments every 3 min, with participants maintaining a sustained cadence of 85–105 rpm, until volitional exhaustion was reached (Beaver et al., 1986; Edvardsen et al., 2014). The maximum rate of O_2_ consumption attained during physical exertion (${\dot{V}}_{O_{2}\max}$) was considered as the highest of the mean values of the rate of O_2_ consumption calculated for the two 30‐s periods that preceded the end of the intervention (Beaver et al., 1986; Edvardsen et al., 2014). Index finger capillary l‐lactate levels before and at the end of the exercise intervention were determined using a Lactate Pro2 portable meter (Arkray, Amstelveen, the Netherlands).

### 2.4 Blood collection and serum preparation and storage

At two time points (15 min before the intervention and within 5 min into active recovery; baseline and post‐intervention serum, respectively), whole blood (10–15 mL) was collected from each participant, immediately transferred to a BD Vacutainer™ SST™ II Advance serum separator tube (Becton Dickinson and Company, Franklin Lakes, NJ, USA) and allowed to clot naturally by leaving it undisturbed at room temperature. After centrifugation (10 min at 1600 *g*), the off‐the‐clot serum was carefully removed and immediately stored at −80°C, in 0.5 mL aliquots, until use. Before use, sera were thawed and, after homogenization, were filtered through 0.2 µm‐pore Millex^®^‐GV sterile syringe filters (Merck, Darmstadt, Germany; SLGV033RB). For each parameter assessed, all sera were tested in parallel.

### 2.5 Quantification of glucose, cholesterol, high‐density lipoprotein cholesterol, low‐density lipoprotein cholesterol, triglycerides and albumin

Reagents from BioSystems (Barcelona, Spain) were used for the quantification of glucose (COD 11504), cholesterol (free and esterified; COD 11505), high‐density lipoprotein cholesterol (HDL cholesterol; COD 11757), low‐density lipoprotein cholesterol (LDL cholesterol; COD 11785), triglycerides (COD 11528) and albumin (COD 11573) in individual sera, according to the manufacturer's instructions. A human, serum‐based general biochemistry calibrator (COD 18044) and human biochemistry control sera (COD 18042 and COD 18043), also from BioSystems, were used for calibration and to verify the accuracy of the measurements, respectively. All analytes were assayed in duplicate, and the two values were averaged.

### 2.6 Quantification of C‐reactive protein

C‐Reactive protein was quantified by turbidimetry using an assay reagent (COD 31321), a calibrator (COD 31113) and a control serum (COD 31213) from Biosystems. All sera were tested in duplicate, and the two values were averaged. The procedure was that specified by the manufacturer.

### 2.7 Culture of A549 cells

All cellular studies were performed in cultures of A549 human lung cancer cells (ATCC CCL‐185). These cells were grown routinely at 37°C in a humidified atmosphere of 5% CO_2_–95% air, in filter‐vented flasks (Orange Scientific, Braine‐l'Alleud, Belgium; 5520100) containing ∼0.2 mL/cm^2^ of growth medium (RPMI‐1640; Merck; R6504) supplemented with 10% (v/v) heat‐inactivated fetal calf serum (FCS; Thermo Fisher Scientific, Waltham, MA, USA; 10270‐106).

To investigate the impact of exercise‐conditioned human serum on cell proliferation, lag phase and plating efficiency, cells were harvested in serum‐free growth medium, centrifuged for 5 min at 200 *g* to remove traces of FCS, and resuspended in an adequate volume of fresh serum‐free medium. A small volume (10 µL) of this serum‐free cell suspension was subsequently added to culture plates already containing prewarmed (37°C) growth medium supplemented with human serum, to a final serum concentration of 10% (v/v).

To investigate the impact of exercise‐conditioned human serum on cell migration and for the determination of half‐maximal cytotoxic concentrations (CC_50_ values) for cisplatin, cells were harvested in growth medium supplemented with 10% (v/v) heat‐inactivated FCS, and new cultures were established in this same medium. At ∼24 h post‐seeding, spent growth medium was replaced by growth medium supplemented with 10% (v/v) human serum.

### 2.8 Assessment of cell proliferation and lag phase

For the assessment of cell proliferation, cultures were prepared in 96‐well plates (Orange Scientific; 4430100), at a seeding density of 5000 cells/cm^2^, in 100 µL of growth medium supplemented with 10% (v/v) human serum. In each independent experiment, a total of six replicate cultures were prepared for each individual serum tested: three each for the estimation of the number of cells in culture at 24 and 72 h post‐seeding. Cell numbers were estimated using the sulphorhodamine B assay (Vichai & Kirtikara, 2006). Absorbance was read at 550 nm (*A*_550_) against a reagent blank (no cells), using a μQuant microplate reader (BioTek Instruments, Winooski, VT, USA). Proliferation rates were then estimated as the fold increase in *A*_550_ over the 48‐h period between the two measurements. The *A*_550_ values at 24 h post‐seeding were used as a metric for lag phase.

### 2.9 Assessment of plating efficiency

Plating efficiencies were evaluated by the clonogenic assay (Franken et al., 2006). To this end, cells were plated as a single‐cell suspension at a colony‐forming density of 40 cells per well in 24‐well plates (Orange Scientific; 4430300), in 500 µL of growth medium supplemented with 10% (v/v) human serum. Each pooled serum was tested in three replicate cultures. After 9 days of incubation, colonies were fixed and stained for 40 min with 400 µL of an aqueous solution containing 6.0% (v/v) glutaraldehyde (Merck; G5882) and 0.5% (w/v) Crystal Violet (Merck; 548629). Afterwards, wells were photographed, colonies were counted manually, and the area of the wells covered by the colonies was measured by ImageJ software v.1.53e (National Institutes of Health, Bethesda, MD, USA). Colony mean size was calculated by dividing this area by the total number of colonies, and plating efficiency was calculated by dividing the number of colonies formed by the number of cells seeded.

### 2.10 Assessment of cell migration

In vitro cell migration was estimated using the wound closure assay (Cappiello et al., 2018; Liang et al., 2007), using silicone three‐well culture inserts (IBIDI^®^, Gräfelfing, Germany; 80369) to create two artificial 500 µm cell‐free gaps (corresponding to two technical replicates) in confluent monolayer cultures. These inserts were placed onto the wells of 24‐well plates (Orange Scientific; 4430300), one insert per well and per individual serum tested. Cells were then seeded into the insert wells, at a density of 2.5 × 10^5^ cells/cm^2^, in 70 µL of growth medium supplemented with 10% (v/v) FCS. After 22 h of incubation, the inserts were removed, monolayers were washed twice with PBS, and three different regions in each gap were imaged (first time point). Prewarmed (37°C) growth medium supplemented with 10% (v/v) human serum (500 µL) was then added, cultures were returned to the incubator and, after 18 h, monolayers were again washed twice with PBS, and the same regions in the gaps were imaged (second time point). All images were captured at 40× magnification, using an Olympus CKX53 inverted optical microscope equipped with a camera and the software EPview™ (v.2.9.6_20201224; Hachioji, Tokyo, Japan). Cell‐free areas in the regions imaged were measured using ImageJ, and the percentage of gap closure over the 18‐h period was calculated. For each serum tested, the average of the respective six percentage values was used as an estimate of cell migration potential.

### 2.11 Determination of CC_50_ values for cisplatin

To determine CC_50_ values for cisplatin, cultures were prepared in 96‐well plates (Orange Scientific), with a seeding density of 5000 cells/cm^2^, in 100 µL of growth medium supplemented with 10% (v/v) FCS. After a 24‐h incubation, spent growth medium was replaced with prewarmed (37°C) growth medium supplemented with 10% (v/v) human serum containing 0–455 µM cisplatin (Merck; 232120), and cultures were incubated for 72 h. For each pooled serum sample, the cytotoxicity of each cisplatin concentration was tested in three replicate cultures using the sulphorhodamine B assay (as described above). The CC_50_ values were obtained from concentration–response curves generated using a non‐linear regression model in GraphPad Prism v.9.0.0. software for Windows (GraphPad Software, Boston, MA, USA).

### 2.12 Semi‐quantitative detection of cytokines

Semi‐quantitative assessment of 80 cytokines was carried out in 0.5 mL samples of pooled sera using the RayBio^®^ C‐Series Human Cytokine Antibody Array C5, from RayBiotech (Norcross, GA, USA; AAH‐CYT‐5‐4), according to the manufacturer's instructions. Samples were diluted 2‐fold with blocking buffer (provided with the kit), and chemiluminescence was detected using a ChemiDoc MP Imaging System (Bio‐Rad, Hercules, CA, USA), after a 30‐s exposure. The signal intensity (average pixels per area) for each antigen‐specific antibody spot was then determined using ImageJ.

### 2.13 Statistical analysis

Data were analysed using GraphPad Prism v.9.0.0. software for Windows. Differences between the two cohorts were assessed for statistical significance using Student's unpaired *t*‐test or the Mann–Whitney *U*‐test, according to the normality of the data, which was verified using the Shapiro–Wilk test. The statistical significance of the differences between the two cohorts in terms of the impact of the acute exercise intervention on growth properties and sensitivity of cells to cisplatin was assessed using repeated‐measures two‐way ANOVA, assuming sphericity. Whenever the null hypothesis was rejected, Šídák's multiple comparison test was performed (Glantz et al., 2001; Maxwell & Delaney, 2004). Differences were considered significant when *P* < 0.05. A *post hoc* analysis, conducted using G*Power (v.3.1.9.4), yielded a power of 0.84 for the repeated‐measures ANOVA (within–between interaction), based on a total sample size of *n* = 26 (*n* = 13 per group), two measurements, an α‐level of 0.05, and an observed effect size *F* = 0.30.

## 3 RESULTS

### 3.1 Cohort characteristics

Selected anthropometric and physiological characteristics of the two age‐matched male cohorts used in this study are summarized in Table 1. The most pronounced anthropometric differences between the cohorts were related to body fat content, with non‐exercisers exhibiting, on average, ∼40% higher fat mass (*P* ≤ 0.001) and 60% higher visceral fat area (*P* = 0.002) than master athletes. Non‐exercisers also exhibited ∼10% lower muscle mass (*P* = 0.008) and 5% higher body mass index (*P* = 0.026).

**TABLE 1:** Anthropometric and physiological characteristics of the two age‐matched male cohorts used in the present study.

Characteristics	Non‐exercisers (*n* = 13)	Master athletes (*n* = 13)	Statistical significance (*P*‐value)
Age, years	49 ± 6	53 ± 7	0.155
Height, cm	172.6 ± 5.7	179.6 ± 9.4	**0.031**
BMI, kg/m^2^	26.7 ± 2.5	25.3 ± 2.7	**0.026**
Muscle mass, %	42.3 ± 2.7	45.7 ± 4.4	**0.008**
Fat mass, %	24.7 ± 5.0	17.6 ± 4.8	**≤0.001**
Visceral fat area, cm^2^	89.2 ± 27.3	56.1 ± 16.7	**0.002**
Basal metabolic rate, kcal/day	1662.0 ± 152.6	1760.0 ± 185.3	0.168
Blood lactate levels at rest^a^, mmol/dm^3^	1.9 ± 0.6	1.7 ± 0.5	0.531
Blood lactate levels post‐intervention^a^, ^b^, mmol/dm^3^	12.7 ± 2.8	11.1 ± 3.0	0.175
${\dot{V}}_{O_{2}\max}$, mL/min/kg	31.7 ± 5.0	39.6 ± 5.6	**≤0.001**
Duration of the exercise intervention^b^, min	13.4 ± 3.3	20.7 ± 4.1	**≤0.001**
Maximum power output^b^, W	170.0 ± 34.8	232.5 ± 35.5	**≤0.001**

*Notes*: Values represent the mean ± SD for the 13 participants in each cohort, except for capillary blood lactate levels, for which values from some participants were excluded for technical reasons (one non‐exerciser and three master athletes, for levels at rest; one master athlete for post‐intervention values). Comparisons between the two cohorts were made using either Student's unpaired *t*‐test (age, height, fat mass, visceral fat, blood lactate levels, ${\dot{V}}_{O_{2}\max}$, duration of the exercise intervention and maximum power output) or the Mann–Whitney *U*‐test (BMI, muscle mass and basal metabolic rate), according to the normality of data, which was assessed using the Shapiro–Wilk test. Statistical significance was set at *P* < 0.05 and is identified in bold type. Abbreviations: BMI, body mass index; ${\dot{V}}_{O_{2}\max}$, maximum rate of O_2_ consumption attained during physical exertion.

^a^ Index fingertip capillary l‐lactate levels.

^b^ The intervention was stopped when the participant reached volitional exhaustion or when at least two of the following three criteria were met: (i) no increase in oxygen consumption despite workload increase; (ii) respiratory exchange ratio > 1.10; (iii) heart rate >90% of the estimated maximal value.

As expected, master athletes exhibited significantly higher cardiorespiratory fitness than non‐exercisers (Rogers et al., 1990; Mckendry et al., 2018), as assessed by the ${\dot{V}}_{O_{2}\max}$ attained during physical exertion and by the duration of the exercise intervention, whose mean values were, respectively, ∼25% (*P* ≤ 0.001) and 50% higher (*P* ≤ 0.001) than those of non‐exercisers. In line with the increase in the duration of the intervention, the maximum power output increased by ∼35% (*P* ≤ 0.001). Basal metabolic rate and blood lactate levels (at rest and after the exercise intervention), in contrast, were not significantly different between the two cohorts. However, the increase in blood lactate levels produced by the exercise intervention differed substantially amongst the different participants in each cohort, with individual increases ranging between 5.2‐ and 9.5‐fold amongst non‐exercisers and between 2.5‐ and 12.1‐fold amongst master athletes (data not shown). These differences were not correlated with either the duration of the exercise intervention or the maximal power output or ${\dot{V}}_{O_{2}\max}$ (Pearson's determination coefficients for these hypothetical correlations, *R* ^2^, ranged between 0.004 and 0.126, and the corresponding *P*‐values ranged between 0.081 and 0.780). In terms of C‐reactive protein (results not shown), a biomarker of inflammation (Sproston & Ashworth, 2018), only one non‐exerciser had a slightly increased baseline serum level (0.72 mg/dL).

None of the small differences between cohorts in the serum levels of glucose, lipids and albumin were statistically significant (Table 2). Also, the effect of the exercise intervention on these levels was equivalent for all analytes tested (all increased by ∼10% in both cohorts; results not shown). As such, rather than a specific response to acute exercise, the increase might reflect a transient small reduction of plasma volume attributable to the intervention (Hansen, 1968).

**TABLE 2:** Baseline serum levels of glucose, cholesterol, triglycerides and albumin in the two age‐matched cohorts used in the present study.

Analyte	Non‐exercisers (*n* = 13)	Master athletes (*n* = 13)	Statistical significance (*P*‐value)
Glucose, mg/dL	104.7 ± 24.6	97.6 ± 17.0	0.605
Cholesterol, mg/dL	212.6 ± 32.4	218.1 ± 29.6	0.654
HDL cholesterol, mg/dL	49.7 ± 12.2	57.4 ± 7.5	0.065
LDL cholesterol, mg/dL	126.3 ± 27.4	123.4 ± 22.3	0.797
Triglycerides, mg/dL	128.6 ± 34.0	106.4 ± 40.5	0.143
Albumin, g/L	50.2 ± 7.4	52.1 ± 2.0	0.373

*Note*: Each serum was tested twice for the different analytes, and the two results were averaged. Values represent the mean ± SD for the 13 participants in each cohort. Comparisons between the two cohorts were made using either Student's unpaired *t*‐test (cholesterol, HDL cholesterol, LDL cholesterol, triglycerides and albumin) or the Mann–Whitney *U*‐test (glucose), according to the normality of data, which was assessed using the Shapiro–Wilk test. For all analytes tested, no statistically significant differences were found between the two cohorts (statistical significance was set at *P* < 0.05). Abbreviations: HDL, High‐density lipoprotein; LDL, Low‐density lipoprotein.

### 3.2 Serum conditioning by acute exercise reduced the proliferation rate of A549 cells, whereas conditioning by lifelong exercise had the opposite effect

**FIGURE 1:**
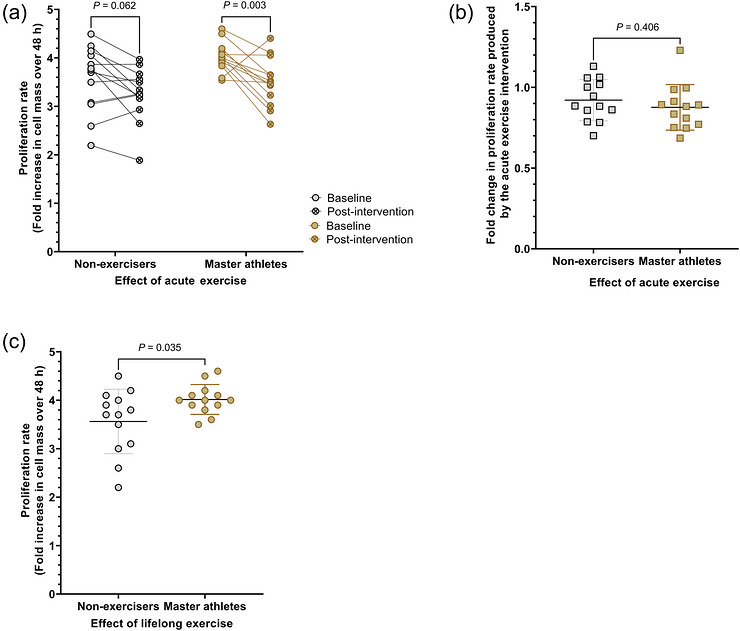
Serum conditioning by acute and lifelong exercise had opposite effects on the proliferation rate of A549 human lung cancer cells. (a) For most of the participants in both cohorts, conditioning by acute exercise reduced the ability of sera to stimulate cell proliferation. (b) On average, the impact of the acute exercise intervention on the ability of sera to stimulate cell proliferation did not differ significantly between the two cohorts. (c) On average, cells stimulated by baseline serum from master athletes exhibited higher proliferation rates than those stimulated by baseline serum from non‐exercisers. All cultures were established and grown in medium supplemented with 10% (v/v) human serum. The number of cells in culture at 24 and 48 h post‐seeding, used to calculate proliferation rates, was estimated using the sulphorhodamine B assay. Circles and squares represent individual values for the different sera and are the means of three independent experiments. In each of these experiments, all conditions (sera and time points) were tested in three replicate cultures. Each set of connected circles represents sera collected from the same participant before (left; baseline) and after (right; post‐intervention) the acute exercise intervention. Large horizontal bars and associated smaller horizontal (error) bars represent the means ± SD for the 13 participants in each cohort. The statistical significance of the effect of lifelong exercise was assessed using Student's unpaired *t*‐test. All other comparisons were made by repeated‐measures two‐way ANOVA, assuming sphericity. Whenever the null hypothesis was rejected, Šídák's multiple comparison test was performed. Statistical significance was set at *P* < 0.05.

As can be appreciated in Figure 1, acute and lifelong exercise had opposite effects on cell proliferation. Serum conditioned by acute exercise decreased cell proliferation by ∼10% (*P* = 0.062) for non‐exercisers and by ∼15% (*P* = 0.003) for master athletes, whereas serum conditioned by lifelong exercise stimulated it by ∼10% (*P* = 0.035). The effect of acute exercise was highly consistent in the cohort of master athletes, being observed in 11 of the 13 (85%) individuals, but less consistent in the cohort of non‐exercisers, where it was observed in only 8 of the 13 (62%) individuals. Nonetheless, the decrease in proliferation rate produced by the intervention did not differ, on average, significantly between the two cohorts (*P* = 0.406).

### 3.3 Serum conditioning by acute exercise increased the lag phase of A549 cells, whereas conditioning by lifelong exercise had the opposite effect

**FIGURE 2:**
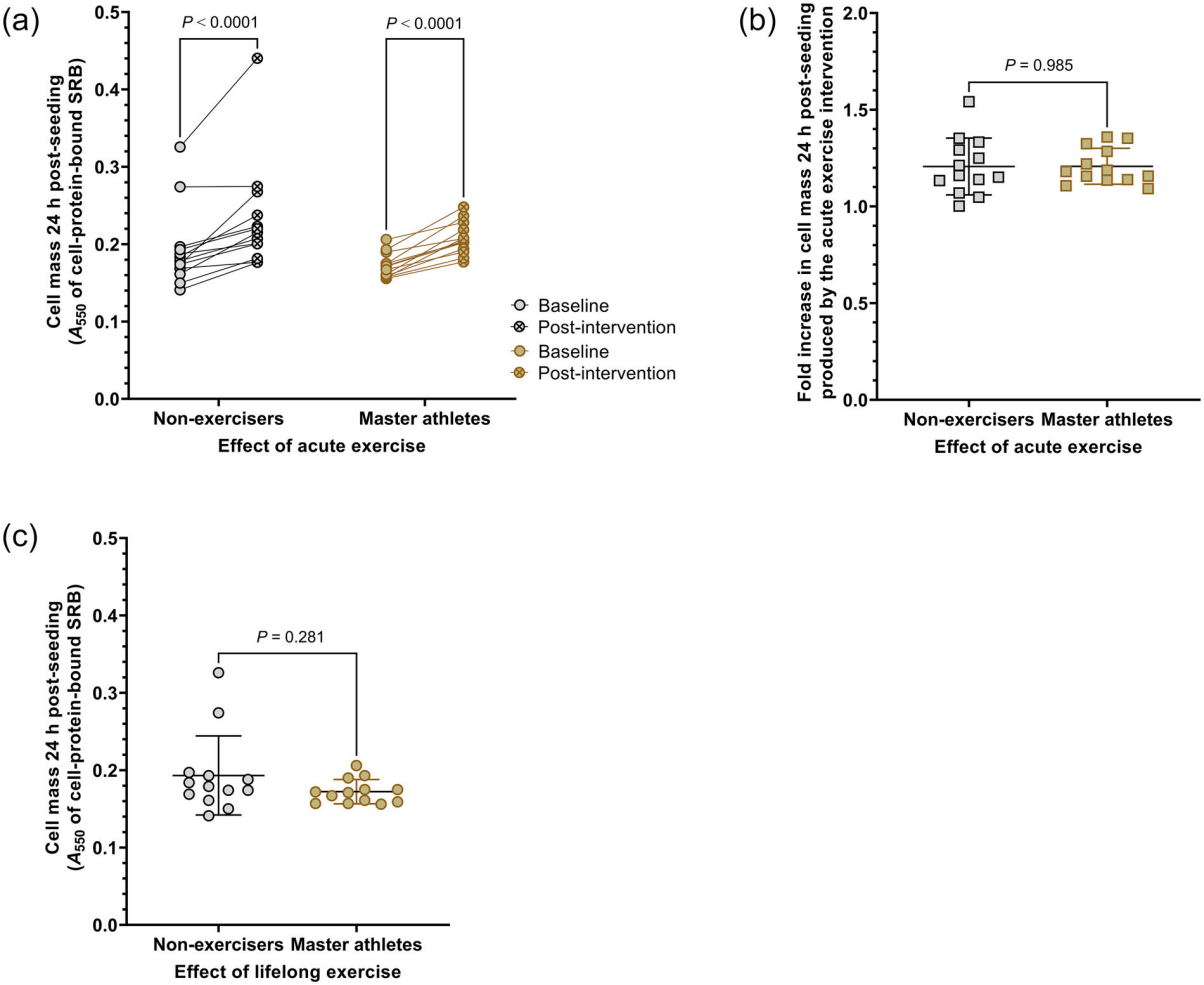
Serum conditioning by lifelong and acute exercise had opposite effects on the number of A549 human lung cancer cells in culture 24 h post‐seeding. (a) For both cohorts, cultures grown in medium supplemented with serum conditioned by the acute exercise intervention (post‐intervention serum) contained, on average, ∼20% more cells 24 h post‐seeding than those grown in medium supplemented with baseline serum from the same participant, indicating a shortened lag phase. This increase in cell number was observed in 12 of 13 (92%) non‐exercisers and in all (100%) master athletes. (b) No statistically significant differences were obtained between the two cohorts in terms of the impact of acute exercise serum conditioning on the number of cells in culture 24 h post‐seeding. (c) On average, cultures grown in medium supplemented with baseline serum from master athletes contained ∼10% less cells 24 h post‐seeding than those grown in medium supplemented with baseline serum from non‐exercisers. All cultures were established and grown in medium supplemented with 10% (v/v) human serum. The total amount of cell protein, assessed using the sulphorhodamine B assay, was used as an estimate of the number of cells in culture. Circles and squares represent individual values for the different sera and are the means of three independent experiments. In each of these experiments, all sera were tested in three replicate cultures, and the values were averaged. Each set of connected circles represents baseline (left) and post‐intervention (right) sera from the same participant. Large horizontal bars and associated smaller horizontal (error) bars represent means ± SD for the 13 participants in each cohort. The statistical significance of the effect of lifelong exercise was assessed using the Mann–Whitney *U*‐test. All other comparisons were made by repeated‐measures two‐way ANOVA, assuming sphericity. Whenever the null hypothesis was rejected, Šídák's multiple comparison test was performed. Statistical significance was set at *P* < 0.05. Abbreviation: *A*_550_, absorbance at 550 nm.

For both cohorts, the number of cells in culture 24 h post‐seeding was, on average, ∼20% higher in cultures stimulated by post‐intervention serum than by the respective baseline serum (*P* ˂ 0.0001 for both cohorts; Figure 2a,b), an effect that was observed in 12 of 13 (92%) non‐exercisers and in all (100%) master athletes. These differences in the number of cells cannot be explained by different proliferation rates, because our proliferation data point in the opposite direction (i.e., cultures with a higher number of cells 24 h post‐seeding had lower proliferation rates; Figure 1). Also, microscopic observation of the cultures did not reveal any differences in the number of dead or unattached cells. Therefore, cells exposed to sera conditioned by acute exercise took less time to attach to the substrate, spread and/or resume proliferation than those exposed to the corresponding baseline sera. No statistically significant effect on the lag phase was observed for cultures exposed to serum conditioned by lifelong exercise (*P* = 0.281; Figure 2c).

### 3.4 Neither acute nor lifelong exercise serum conditioning affected the reproductive potential or the migratory capacity of A549 cells

**FIGURE 3:**
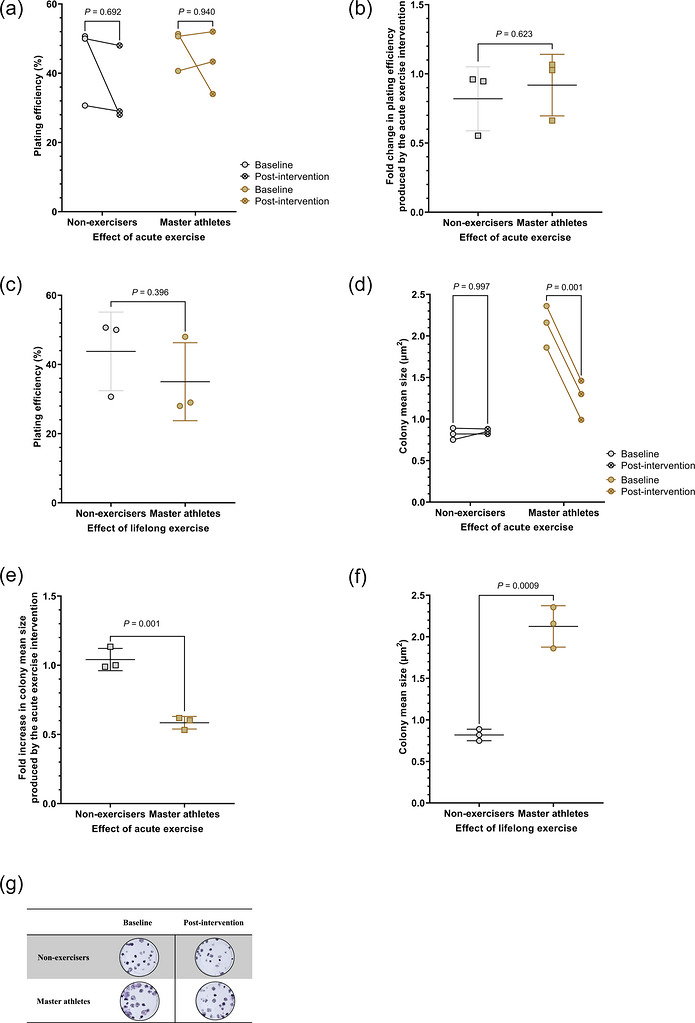
Neither acute nor lifelong exercise serum conditioning affected the reproductive potential of A549 human lung cancer cells, as assessed by their plating efficiencies. Pooled sera were used for the four conditions tested (baseline non‐exercisers; baseline master athletes; post‐intervention non‐exercisers; and post‐intervention master athletes), each containing equal volumes of serum from 13 participants. (a–c) Plating efficiencies, expressed as percentage values, were calculated by dividing the number of colonies formed by the number of cells seeded. (d–f) Colony mean sizes were calculated by dividing the area covered by the colonies (measured using ImageJ) by the total number of colonies. Each set of three circles or squares represents the values obtained in three independent experiments. In each of these experiments, all pooled sera were tested in three replicate cultures and the results averaged. Large horizontal bars and associated smaller horizontal (error) bars represent means ± SD for the three independent experiments. The statistical significance of the effect of lifelong exercise was assessed using Student's unpaired *t*‐test. All other comparisons were made by repeated‐measures two‐way ANOVA, assuming sphericity. Whenever the null hypothesis was rejected, Šídák's multiple comparison test was performed. Statistical significance was set at *P* < 0.05. No statistically significant differences were obtained: (i) in plating efficiency between baseline and corresponding post‐intervention pooled sera in the two cohorts and between baseline sera from the two cohorts; or (ii) in the mean size of the colonies between baseline and post‐intervention sera in non‐exercisers. (g) Representative photographs of the colonies that formed after 9 days of incubation when cells were plated using a single‐cell suspension at a colony‐forming density of 40 cells per well in 24‐well plates, in 500 µL of growth medium supplemented with 10% (v/v) of pooled human serum.

Our results show that neither acute exercise nor lifelong exercise had a significative impact on plating efficiency, used here as a metric for reproductive potential [Figure 3a–c; *P* = 0.692 (non‐exercisers) and *P* = 0.940 (master athletes) for the effect of acute exercise, and *P* = 0.396 for the effect of lifelong exercise]. Regarding the very large changes in colony size observed (Figure 3d–f), they are likely to be a result of the alterations in proliferation rates discussed above (Figure 1). It must be noted that small differences in proliferation rate, such as those observed in our study, can translate into markedly different numbers of cells at the end of 9 days (the duration of the assay used).

**FIGURE 4:**
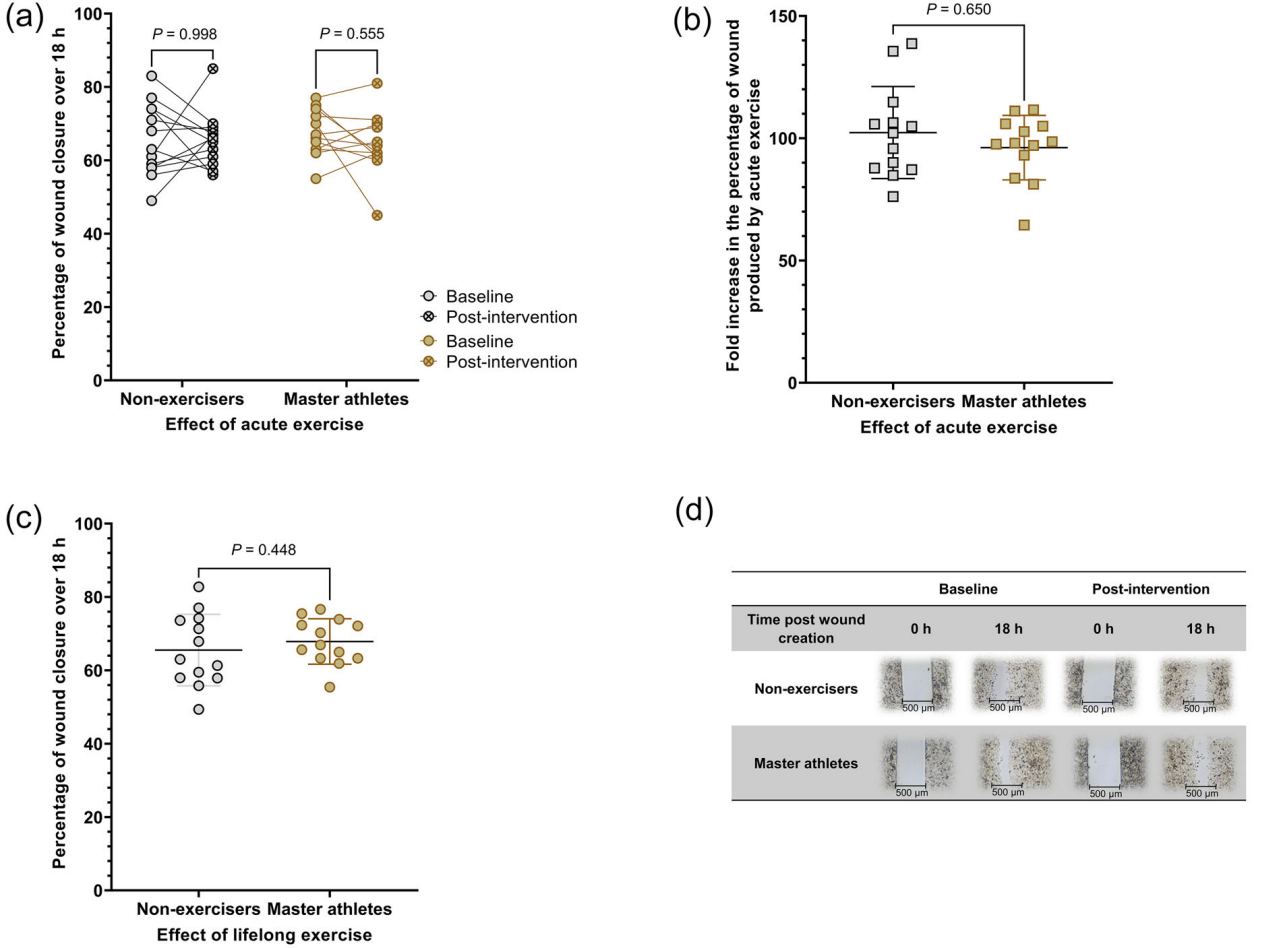
Neither acute nor lifelong exercise serum conditioning affected the in vitro migratory capacity of A549 human lung cancer cells. (a–c) To assess in vitro migratory capacity, two artificial 500 µm cell‐free gaps were created in confluent monolayer cultures, using three‐well culture inserts that were placed onto the wells of 24‐well plates, one insert per well and per serum sample tested. Cultures were then exposed to medium supplemented with 10% (v/v) human serum. For each gap, three different regions were imaged immediately after its creation and after an 18 h incubation. Cell‐free areas in these images were then measured using ImageJ, and the percentage of gap closure over this period was calculated. For each serum, the average of the corresponding six values was then used as an estimate of cell migration potential. Circles and squares represent individual values for the different sera. Each set of connected circles represents baseline (left) and post‐intervention (right) sera from the same participant. Large horizontal bars and associated smaller horizontal (error) bars represent means ± SD for the 13 participants in each cohort. The statistical significance of the effect of lifelong exercise was assessed using the Mann–Whitney *U*‐test. All other comparisons were made by repeated‐measures two‐way ANOVA, assuming sphericity. Statistical significance was set at *P* < 0.05, and the null hypothesis was never rejected. Results are from a single independent experiment, in which each serum was tested in two artificial gaps. (d) Representative micrographs (40× magnification) of artificial gaps for each of the four conditions tested, taken immediately after the creation of the gap and after an 18‐h incubation in growth medium supplemented with 10% (v/v) human serum. Micrographs were captured using an Olympus CKX53 inverted optical microscope equipped with a camera and the EPview™ software (v.2.9.6_20201224; Hachioji, Tokyo, Japan).

As can be appreciated in Figure 4, neither acute nor lifelong exercise conditioning altered the ability of serum to promote cell migration to a statistically significant extent [Figure 4a–c; *P* = 0.998 (non‐exercisers) and *P* = 0.555 (master athletes) for the effect of acute exercise, and *P* = 0.448 for the effect of lifelong exercise]. It must be noted, however, that cells were exposed to human sera for a relatively short period (18 h). Also, owing to the limited volume of human serum available, the results are from a single experiment (in which all 52 sera were tested in duplicate).

### 3.5 Lifelong exercise increased the sensitivity of A549 cells to cisplatin

**FIGURE 5:**
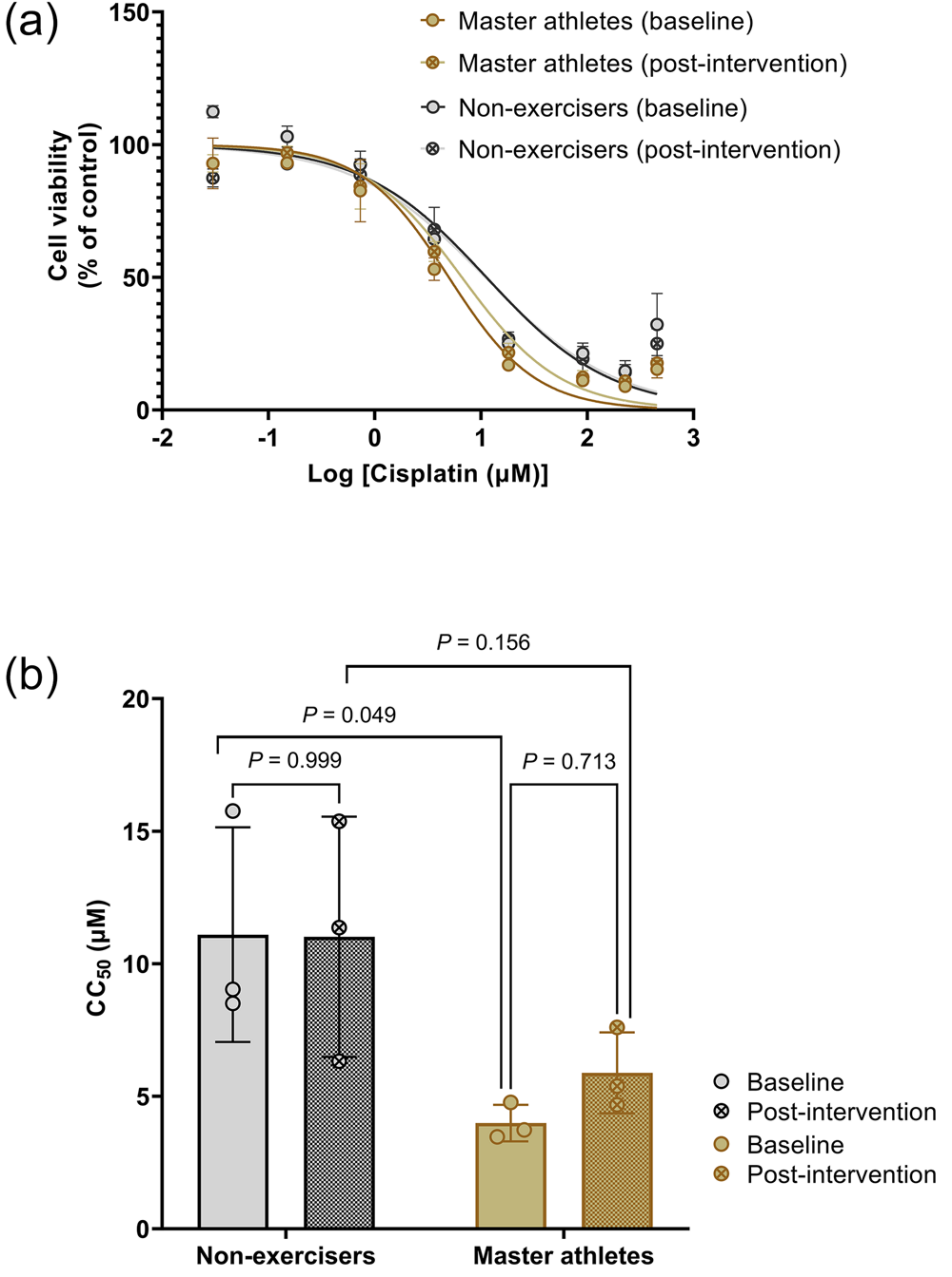
Lifelong exercise more than doubled the sensitivity of A549 human lung cancer cells to cisplatin. (a) Concentration–response curves depicting the cytotoxicity of cisplatin against cells stimulated with pooled sera (10% v/v) from non‐exercisers or master athletes, obtained at rest (baseline) or after an acute exercise intervention (post‐intervention). (b) CC_50_ values for cisplatin for cultures incubated with the four different pooled sera. Cell viability was estimated at 72 h post‐cisplatin addition using the sulphorhodamine B assay. For each pooled serum, the impact of the different cisplatin concentrations (0–455 µM) on cell viability was tested in three independent experiments, each with three replicate cultures per concentration, and expressed as percentage of the control value (0 µM cisplatin). CC_50_ values were calculated from concentration–response curves generated using a non‐linear regression model in GraphPad Prism. Circles and corresponding error bars represent means ± SD, respectively, for the three independent experiments. Each pooled serum contained equal volumes of serum from 13 participants. Comparisons between the indicated groups were made by: (a) the sum‐of‐squares using the extra sum‐of‐squares *F*‐test; or (b) Student's unpaired *t*‐test (effect of lifelong exercise) and repeated‐measures two‐way ANOVA, assuming sphericity (effect of acute exercise). Whenever the null hypothesis was rejected, Šídák's multiple comparison test was performed. Statistical significance was set at *P* < 0.05. Abbreviation: CC_50_, half‐maximal cytotoxic concentration.

As can be appreciated in Figure 5, sensitivity to cisplatin was much higher (as assessed by the lower CC_50_ value) when cells were incubated in the presence of baseline serum from master athletes than baseline serum from non‐exercisers (*P* = 0.04). On the contrary, acute exercise had no significant impact on this sensitivity (*P* = 0.999 and *P* = 0.712, for non‐exercisers and master athletes, respectively).

### 3.6 Both acute and lifelong exercise modulated serum cytokine levels

**FIGURE 6:**
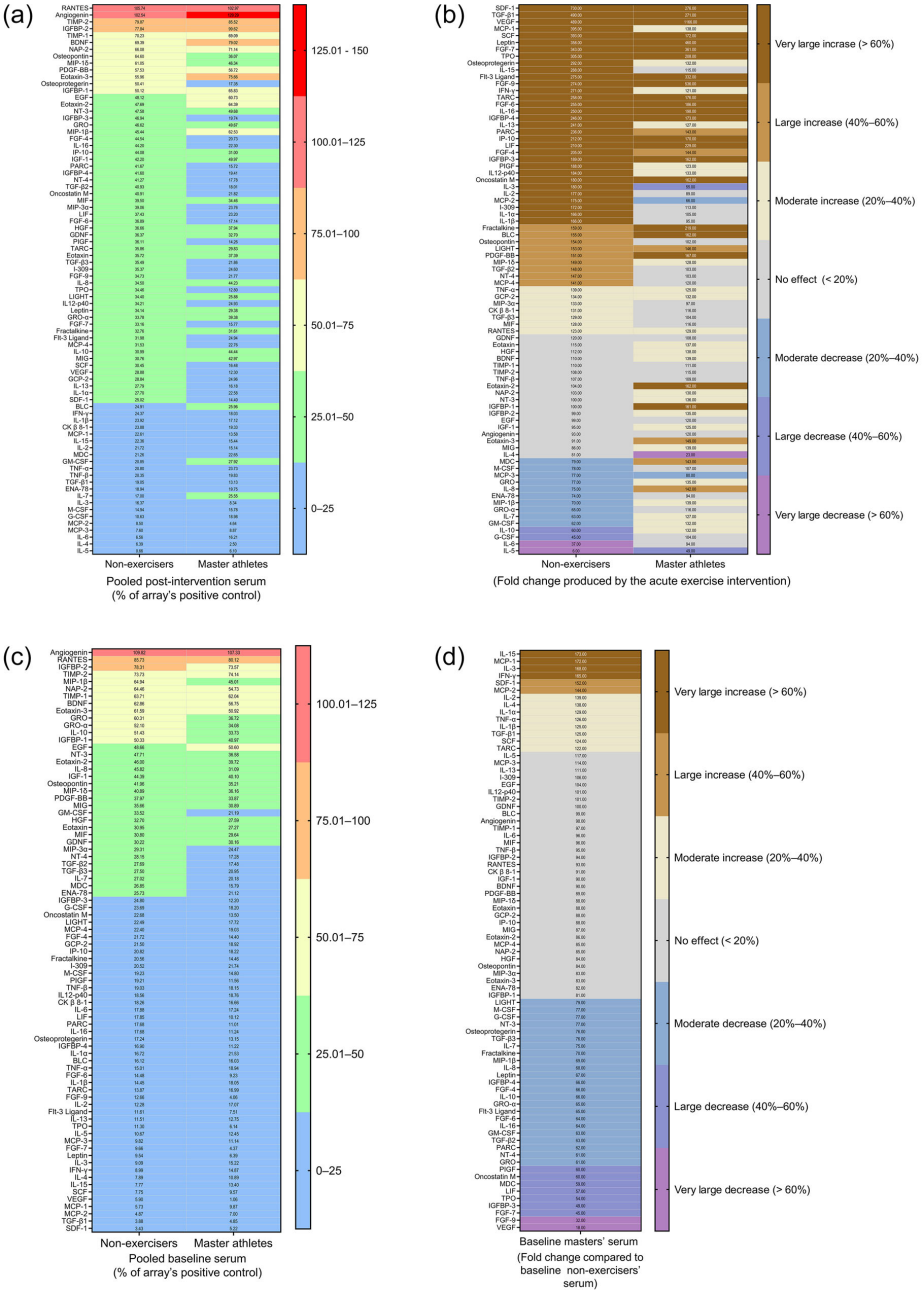
The effects of exercise on serum cytokine levels depended on the cytokine, the duration of exercise (acute vs. lifelong) and previous levels of physical activity (non‐exercisers vs. master athletes). Semi‐quantitative assessment of the 80 cytokines was carried out in pooled sera, each containing equal volumes of serum from 13 participants, using an antibody‐based microarray. For each antigen‐specific antibody spot, chemiluminescence signal intensity (average pixel/area) was determined using ImageJ. Two independent determinations were carried out, and the results were averaged (a, c) Cytokine levels are presented in decreasing order of signal intensity in the pooled serum from non‐exercisers. (b, d) Cytokines are presented in order of fold change in signal intensity produced by the acute exercise intervention in the serum from non‐exercisers.

Acute exercise increased the levels of most cytokines (∼50 in both cohorts), and these increases tended to be substantial (up to 7‐fold). Unlike in serum from non‐exercisers, where acute exercise produced a very large increase in interleukin‐1 beta (IL‐1β), interleukin‐2 (IL‐2) and interleukin‐15 (IL‐15), among others, and a very large decrease in interleukin‐6 (IL‐6), among others, no effect was observed in the levels of these cytokines in serum from master athletes. Interestingly, for some cytokines, such as the growth‐regulated oncogene (alpha/beta/gamma) (GRO (α/β/γ), interleukin‐8 (IL‐8; CXCL8), interleukin‐10 (IL‐10) and macrophage inflammatory protein‐1 beta (MIP‐1β; CCL4), acute exercise had opposite effects in the two cohorts, reducing their levels in non‐exercisers, but augmenting them in master athletes (Figure 6a).

Regarding lifelong exercise, it had no effect on ∼40% of the cytokines tested, and when it did have an effect it was mostly a decrease: 32 cytokines saw their signal intensity decrease. The 14 cytokines that increased, most notably monocyte chemoattractant protein 2 (MCP2), stromal cell‐derived factor 1 (SDF1) and transforming growth factor beta 1 (TGF‐β1 (Figure 6b), displayed very weak signals, near the limit of detection, in both cohorts, and their increase might thus not represent true physiological changes. On the contrary, many of the cytokines whose signals were significantly reduced in the serum of master athletes compared with the serum of non‐exercisers, namely granulocyte‐macrophage colony‐stimulating factor (GM‐CSF), GRO (α/β/γ), GRO‐α, IL‐8, IL‐10, MIP‐1β, neurotrophin‐3 (NT‐3) and osteopontin, were significantly expressed in both cohorts.

## 4 DISCUSSION

The aim of the present investigation was 3‐fold: (i) to strengthen the evidence regarding the impact of serological responses to exercise on the proliferation and reproductive potential of cultured human cancer cells; (ii) to expand this line of investigation to cell migration, a cell property also frequently used as a marker of transformation degree and, ultimately, tumorigenic potential, which has not been investigated previously in this type of study (compromising or losing any of these markers might ultimately halt or reverse cancer progression); and (iii) to explore, for the first time, whether these serological responses might alter the sensitivity of cancer cells to chemotherapy agents. Our study used two cohorts of age‐matched individuals who differed significantly in terms of levels of physical activity, specifically individuals who did not meet the WHO guidelines on physical activity and master athletes (as a model of lifelong exercise). The simultaneous use of two distinct cohorts allowed us to gain insight into the dependence of the cellular effects induced by acute exercise on cohort characteristics. All cell studies were carried out on cultures of A549 cells, which were used as an in vitro model of human lung cancer, for which epidemiological evidence linking physical activity with reduced cancer risk is strong (Moore et al., 2016).

Our proliferation data (Figure 1a,b) support previous findings that acute exercise decreases cancer cell proliferation (Baldelli et al., 2020; Dethlefsen et al., 2016, 2017; Devin et al., 2019; Hwang et al., 2020; Kim et al., 2023; Kurgan et al., 2017; Orange et al., 2022; Rundqvist et al., 2013). Lifelong exercise, on the contrary, increased cell proliferation (Figure 1c). This contrasts with the reports that 10+ years of training decreased the proliferation of LNCaP prostate cancer cells and did not alter the proliferation of an LNCaP‐derived cell line with non‐functional p53 (LN‐56 cells), suggesting that the effects on cell proliferation depend on the characteristics of the cells, e.g., their p53 status (Barnard et al., 2003; Leung et al., 2004). There are also reports of reduced proliferation when cancer cells were stimulated by sera from cancer patients conditioned by short‐term (3–6 months) exercise programmes (Bettariga et al., 2026; Kim et al., 2022a, 2022b). On the contrary, in all three studies that assessed the effects of both short‐term exercise programmes (4–9 weeks) and acute exercise, it was found that the former did not affect cell proliferation, whereas the latter decreased it (Baldelli et al., 2020; Dethlefsen et al., 2016; Devin et al., 2019; Kim et al., 2022a). This contrasting pattern led the authors of these three studies to hypothesize that the putative beneficial effects of long‐term exercise training on cancer incidence, recurrence and survival result from the cumulative effects of transient serum changes in response to each repeated exercise bout, rather than from permanent alterations to serum produced by exercise training (Baldelli et al., 2020; Dethlefsen et al., 2016; Devin et al., 2019). However, it must be stressed that the duration of exercise programmes might not have been sufficient to produce permanent changes in serum to factors affecting cell proliferation.

A contrasting pattern was also found in terms of the impact of the two different types of exercise on the lag phase, with acute exercise decreasing it (Figure 2a,b) and lifelong exercise extending it (Figure 2c). Given that the present study is the first to investigate the effects of exercise on this property, it is not possible to tell whether this effect is specific to the A549 cell line and/or the cohorts used, or whether it is a more general one. It is known that interactions between cells and the extracellular matrix modulate cell survival and proliferation (Frantz et al., 2010). However, considering the limited evidence available, it would not be advisable to speculate on possible consequences of such an effect on the tumorigenic potential of the cells. Also, when it comes to cancer, adhesion to the extracellular matrix can be seen as a double‐edged sword. In fact, in order to metastasise, cancer cells must initially detach from neighbouring cells and the extracellular matrix, then resist anoikis during their migration to other parts of the body. But they then need to reattach and resume proliferation to form tumours in new locations (Shaw et al., 2025; Welch & Hurst, 2019).

Neither acute exercise (Figure 3a,b) nor lifelong exercise (Figure 3c) produced significant changes in plating efficiency, indicating that the reproductive potential was unchanged. This finding contrasts with a previously reported significant decrease in the number of colonies formed by A549 and two other cell lines when sera were conditioned by acute exercise (Kurgan et al., 2017). Once again, these distinct outcomes might be attributable to differences in study design, including the criteria used for counting colonies.

Together with cell–cell adhesion and cell adhesion to the substrate, the migratory capacity is often used as a metric for metastatic potential (Mehanna et al., 2025). In our study, neither acute exercise (Figure 4a,b) nor lifelong exercise (Figure 4c) altered the migratory capacity of A549 cells. However, cells were exposed to human serum for a relatively short period, and it cannot be excluded that a longer exposure could have produced a different outcome.

A most striking result of our study was the strong impact that lifelong exercise had on the sensitivity to cisplatin, which more than doubled (Figure 5). Thus, one of the mechanisms by which exercise might decrease cancer mortality, besides improved immunosurveillance (Bigley et al., 2014; Moro‐García et al., 2014), is by increasing the cytotoxicity of chemotherapeutic agents against cancer cells, thus improving therapeutic efficacy for patients. This is the first report of this type of effect and, owing to its significance, this approach should undoubtedly be pursued.

Aiming to shed light on the molecular mechanisms behind the distinct impacts of acute and lifelong serum conditioning on the behaviour of cancer cells, we interrogated cytokine levels in sera of non‐exercisers and master athletes at baseline and after acute exercise (Figure 6). It is well known that skeletal muscle acts as an endocrine organ that produces and secretes hundreds of cytokines and other signalling peptides in response to exercise. Collectively known as myokines, these molecules mediate communication within skeletal muscle and between skeletal muscle and other organs, having a wide array of functions (Bettariga et al., 2025; Caan et al., 2018; Pedersen & Febbraio, 2012; Severinsen & Pedersen, 2020). Myokines, such as decorin, irisin, oncostatin M and secreted protein acidic and rich in cyeteine (SPARC), have been implicated in the preventive and therapeutic effects of exercise and can directly affect cancer cell behaviour by several means, such as by inhibiting proliferation, promoting apoptosis and inhibiting epithelial‐to‐mesenchymal cell transition, ultimately limiting invasion and metastasis (Huang et al., 2022; Kim et al., 2021).

Direct comparisons of the present work with other studies or between our two cohorts are not straightforward, because it has been found that exercise‐mediated cytokine secretion depends on the characteristics of the exercise, such as the type, intensity and duration (Kim et al., 2021; Piccirillo, 2019). Also, cytokine serum levels are notoriously low (in the picomolar or femtomolar range) (Anderson & Anderson, 2002). Accordingly, chemiluminescent signals for some cytokines were very weak. Nonetheless, some comments can be made tentatively. The effects on the serum levels of many cytokines were dependent on the type of exercise (acute vs. lifelong) and cohort, as was the case with the observed effects on cell growth characteristics. Also, most of the cytokines that were affected by acute exercise had their levels increased, whereas the opposite was true for lifelong exercise. Of note, despite the changes it induced, lifelong exercise did not substantially alter the pattern of signal intensity; cytokines present at high levels in non‐exercisers were also highly expressed in master athletes, and the same was true for minimally expressed cytokines. Globally speaking, the increases produced by acute exercise were more pronounced in non‐exercisers than in the master athletes. For some cytokines, such as IL‐8, GRO (α/β/γ) and MIP‐1β, chemokines responsible for recruiting neutrophils and monocytes to sites of inflammation (Fujiwara et al., 2002; Parekh et al., 2019), and IL‐10, responsible for the anti‐inflammatory effect of exercise (Gleeson et al., 2011), acute exercise had opposite effects in the two cohorts, reducing their serum levels in non‐exercisers, but augmenting them in master athletes. Of note, serum levels of several cytokines linked to the senescence‐associated secretory phenotype (Li et al., 2023) were lower in master athletes than in non‐exercisers, namely those of eotaxin‐3 (CCL26), GM‐CSF, GRO, IL‐8, MIP‐1β and macrophage inflammatory protein 1 delta (MIP‐1δ). Upon acquisition of this phenotype, fibroblasts become pro‐inflammatory cells with the ability to promote tumour progression (Coppé et al., 2010).

### 4.1 Limitations

Cellular studies are prone to a high degree of variability, owing to heterogeneity within cell lines. To increase the robustness of our data, multiple biologically independent experiments were carried out, and each serum sample was tested in at least two technical replicates for each of these experiments. Given that the volume of blood collected was limited, for ethical considerations, pooled sera, instead of individual sera, were used in the assessment of some parameters, an approach that eliminates inter‐individual variability. In addition, the study was conducted on a single cell line.

Our study is the first to address the impact of exercise on the susceptibility of cultured cancer cells to a chemotherapy drug (cisplatin) and on a series of properties that characterize the growth pattern of cultured human cancer cells, i.e., migratory capacity and cell adhesion to the substrate, resumption of cell growth and division after detachment. Other strengths of our study include the parallel investigation of the effects of both transient and permanent serum responses to acute and lifelong exercise, respectively. Of note, we are the first to examine master athletes in this type of study and to assess, in parallel, the effects of acute exercise on two cohorts differing significantly in their baseline exercise levels.

## 5 CONCLUSION

In summary, we found, for both cohorts, that acute exercise decreased the proliferation rate of A549 cells, as previously reported by several groups for this and other cell lines. Interestingly, although acute exercise decreased the proliferation rate, it shortened the time that cells took to adhere to the substrate, spread and resume proliferation. Lifelong exercise had the opposite effects; it increased the proliferation rate and increased the lag phase. Regarding plating efficiency and migratory capacity, no effect could be detected. A different outcome for migratory capacity might have been observed if the duration of the exposure to human serum had been increased. Strikingly, lifelong exercise more than doubled the sensitivity of cancer cells to cisplatin, which might explain, together with other mechanisms, such as enhanced immunosurveillance, the lower cancer mortality rates found among those who exercise regularly. Moreover, serum cytokine patterns induced by lifelong exercise and acute exercise contrasted sharply, potentially contributing to the different impacts that these two types of exercise had on cancer cell properties.

## AUTHOR CONTRIBUTIONS

Carlos M. Soares: conceptualization, investigation, formal analysis, resources, writing—review, editing, and visualization. João P. Moura: investigation, formal analysis, writing—review, and editing. Leonardo M. R. Ferreira: conceptualization, writing—review, and editing. Ana Pedrosa: resources and writing–review. Pedro Filipe: resources and writing–review. Luís Rama: resources and writing–review. Ana M. Teixeira: conceptualization, resources, writing–review, editing, and supervision. Ana M. Urbano: conceptualization, formal analysis, investigation, resources, writing—original draft, writing–review, editing, supervision, and funding acquisition. All authors have read the final version of the manuscript and agree to be accountable for all aspects of the work in ensuring that questions related to the accuracy or integrity of any part of the work are appropriately investigated and resolved. All persons designated as authors qualify for authorship, and all those who qualify for authorship are listed.

## CONFLICT OF INTEREST

The authors declare that they have no conflicts of interest. The funders had no role in the design of the study; in the collection, analyses or interpretation of data; in the writing of the manuscript: or in the decision to publish the results.

## GENERATIVE AI STATEMENT

No generative AI tools were used in the preparation of this manuscript.

## Data Availability

The datasets generated during the present study are available from the corresponding author upon reasonable request.
